# Lessons learned from IDeAl — 33 recommendations from the IDeAl-net about design and analysis of small population clinical trials

**DOI:** 10.1186/s13023-018-0820-8

**Published:** 2018-05-11

**Authors:** Ralf-Dieter Hilgers, Malgorzata Bogdan, Carl-Fredrik Burman, Holger Dette, Mats Karlsson, Franz König, Christoph Male, France Mentré, Geert Molenberghs, Stephen Senn

**Affiliations:** Department of Medical Statistics, RWTH Aachen University, Pauwelsstr. 19, D-52074 Aachen, Germany

**Keywords:** Statistical methodology, Statistical design, Statistical analysis, Small population clinical trials, Rare disease

## Abstract

**Background:**

IDeAl (Integrated designs and analysis of small population clinical trials) is an EU funded project developing new statistical design and analysis methodologies for clinical trials in small population groups. Here we provide an overview of IDeAl findings and give recommendations to applied researchers.

**Method:**

The description of the findings is broken down by the nine scientific IDeAl work packages and summarizes results from the project’s more than 60 publications to date in peer reviewed journals. In addition, we applied text mining to evaluate the publications and the IDeAl work packages’ output in relation to the design and analysis terms derived from in the IRDiRC task force report on small population clinical trials.

**Results:**

The results are summarized, describing the developments from an applied viewpoint. The main result presented here are 33 practical recommendations drawn from the work, giving researchers a comprehensive guidance to the improved methodology. In particular, the findings will help design and analyse efficient clinical trials in rare diseases with limited number of patients available. We developed a network representation relating the hot topics developed by the IRDiRC task force on small population clinical trials to IDeAl’s work as well as relating important methodologies by IDeAl’s definition necessary to consider in design and analysis of small-population clinical trials. These network representation establish a new perspective on design and analysis of small-population clinical trials.

**Conclusion:**

IDeAl has provided a huge number of options to refine the statistical methodology for small-population clinical trials from various perspectives. A total of 33 recommendations developed and related to the work packages help the researcher to design small population clinical trial. The route to improvements is displayed in IDeAl-network representing important statistical methodological skills necessary to design and analysis of small-population clinical trials. The methods are ready for use.

## Background

IDeAl is an EU funded project aiming to refine the statistical methodology in small-population group trials by strictly following the concept of an improved integration of design, conduct and analysis of clinical trials from various perspectives. The CHMP guidance [1] on small-population clinical trials being published almost 10 years ago, as well as the closely related recent draft guidance on extrapolation set up the scene for IDeAl’s developments over the past 3 years. In particular, the CHMP guidance stated that there exist no specific statistical methods for small population clinical trials. This is in stark contrast to the ambition of the international rare diseases research consortium [2] to foster diagnosis and therapies in rare diseases, which of course is the most prominent application area of small-population clinical trials. The statistical methodological challenges to design and analyse of such trials were recently described [3]. IDeAl addressed the challenges within its nine scientific work-packages: adaptive design, biomarkers, decision theory, extrapolation, genetic factors, optimal design, pharmacogenetics, randomisation and simulation. Although the work-packages at a first glance appear to address disparate methodological issues, the overarching topics are obvious. For example, adaptive “designing” or thinking can obviously be applied in finding an efficient design for a clinical trial where the methodology might also be used to combine several trials given that it may be useful in using external information, as well as in determining the type of evidence looked for. Similarly in randomisation, a technique which is useful in designing a trial in particular as an N-of-1 trial but its implications for the level of evidence derived from a clinical trial has to be considered carefully. Further non-linear mixed effects modelling is not only a useful and well established technique in the pharmacometrical context but also to establish surrogate endpoints. IDeAl has described the findings in currently more than 60 peer-reviewed papers but an applied researcher might be lost in navigating through the results. Furthermore, an applied researcher having a rough idea about possible important aspects to be considered in small-population clinical trials may wonder about options to improve standard design techniques. Thus, the objective of this paper, is to build up an umbrella of IDeAl’s research findings and to give recommendations for design and analysis of small population clinical trials and identify researchers’ “ideas” expressed in topics covered by the IDeAl programme.

### Paper outline

The present paper is structured according to the nine IDeAl scientific work-packages embedded in the directions for new developments [2]. Each section ends up with a set of recommendations. The final section give an IDeAl view, where necessary methodological skills to apply IDeAl’s methods are visualized by the network “IDeAl-net”.

## Level of evidence - decision theory

The question whether a drug is providing benefit to the patients appears in the very early beginning as well at the end of the drug development program.

The final decision to apply a new treatment or drug depends on the level of evidence derived from a set of trials. Above, various methods are discussed to improve the level of evidence of a single trial, whereas the evidence from several trials is usually synthesized by meta-analytic approaches. When focusing on the evidence gathered from sequential analysis of inferences, we showed that the stopping rule does not have an influence on the inferences from a meta-analysis provided that the trials are weighted by information provided [4]. Thus inferences from combining small trials in rare diseases are unaffected by whether the trials were sequential or not.

In the field of development of drugs for small populations, we can do more than improving the statistical methodology for one or a series of trials. In addition to optimized trial designs, we should also consider the decisions that determine whether a new treatment will be coming to the market. As already observed in the pediatric area [5], it could be disappointing to wait on new drug licensing in disease areas with limited populations, in particular under non increasing R&D investment of the pharmaceutical industry [6]. Different stakeholders have to come to positive decisions and their varying opinions should be recognized. This may help to balance the arguments and prepare the ground for new development programs. We follow a decision theoretic way to evaluate the interactions of different decision making stakeholders, and to provide recommendations for regulators, reimbursers and trial sponsors. Commercial drug development is heavily dependent on EU regulations, EMA decisions and national reimbursement decisions. IDeAl has demonstrated that if pharmaceutical companies experience non-transparency in such societal decision rules, such as uncertainty of how benefit/risk and cost/effectiveness are weighted, the industry will not be able to design the best possible trial programs [7]. Given a successful trial, it also models the sponsor’s pricing and the reimburser’s reaction to that. Considering a population of candidate drugs, we lay out the public incentivizing structure, in terms of requirements on clinical evidence, and study the relation to sponsor’s willingness to invest [8]. When a potentially predictive biomarker is present, a model was proposed for how the design of the trial will affect expected public benefit as well as commercial value [9, 10]. Further aspects of adaptations are considered as well. Dosing and sizing is modelled, and a decision-theoretic framework for program optimization is sketched [11]. A pure societal perspective is set up [7], where the goal function is simply to maximize the total health benefit in a limited population. In addition the impact of non-transparency in the regulators’ benefit-risk evaluation on optimal decisions taken by the commercial sponsor was modeled. Regarding regulatory rules, as well as regarding reimbursement rules [7], failure to communicate precise rules to other stakeholders, may lead to suboptimal design and development decisions by sponsors. One recommendation is to increase transparency in regulatory and payer decisions.

A general recommendation is to formulate decision rules in a formal Bayesian decision theoretic framework. Even sub-optimal decisions can be modelled [7], explicitly assessing the uncertainty from one stakeholder’s point of view of how another stakeholder will make decisions in different scenarios.

The methodology used in the work package is based on decision theory. It has a distinct flavor of social science, when addressing policy issues, when discussing the formulation of utilities, and in assumptions about (so called) rational agents. This methodology has also some relevance to the important ethical issues around experimentation in human beings. We find that what is best for a patient, who may be included in a clinical trial, may be quite different from what gives the highest overall societal utility. We argue that the well-being of the individual patient must have priority [9].

Finally, we consider investment decisions. It is perhaps not surprising that we find that rational sponsors prefer to invest in drugs with larger market potential, and that sample sizes also tend to increase. We find that this behavior is partly optimal also from a public health perspective. However, there is often a discrepancy between sponsor and societal optimality. In our model [8], larger sample sizes are generally favored from a public health view. Designs motivated by public health consideration will more often focus on the biomarker positive subpopulation. By applying mechanism design, explicitly considering how regulations will affect sponsor decisions, societal rules can be optimized. In the framework [7, 8], the sample size decreases with lower prevalence of the disease. Also, the regulatory requirements should be tailored to the population size. It is recommended that societal decision rules should be determined based on an understanding, and explicit modelling, of how they will inter-depend with commercial drug developing decisions.

Our research has shown how the expected net present value can be maximized, by tuning design parameters as sample size and trial prevalence. The pricing of a new pharmaceutical has also been optimized [7].

*To summarize, we evaluated how to optimize the overall value of drug development to patients, to regulators and to society under opacity in regulatory and payer rules as well as in very rare diseases.*

1. Formulate decision rules in a formal Bayesian decision-theoretic framework [7].
2. Societal decision rules (regulation, reimbursement) should be determined based on explicit modelling of how they will inter-depend with commercial drug developing decisions [7].
3. Increase transparency in regulatory and payer decisions [8].
4. The well-being of the individual trial patient must have priority [9].

## Pharmacological consideration - simulation

Recently pharmacometrical modelling via application of nonlinear mixed-effects models (NLMEM) [12] attracted recognition as a useful methodology to aid design, sample size determination, endpoint selection, and analysis of clinical trials. Analysis of clinical trial data using NLMEM can provide important advantages both with respect to the type of information gained and the statistical power for making inference [12, 13]. In general, the main disadvantage with a non-linear mixed effects modelling approach is the assumptions needed for the models. However, with the movement towards mechanistic models based on biological understanding [14, 15], the validity of model assumptions becomes easier to evaluate. Mechanism-based NLMEMs can be of special interest in small population groups for multiple reasons [16], like gain in statistical power using as much biological knowledge as possible.

For more complex, longitudinal models the joint distribution of the observations is less obvious and even the effect size might not be easily derivable. In this situation, usually no analytic derivation of the power can be obtained and one has to resort to Monte-Carlo simulations. Ideally, a Monte-Carlo study utilizes a model containing all available knowledge for a particular compound to simulate replicates of the trial and the intended analysis model (not necessarily equivalent to the simulation model) to analyse these replicates. A novel parametric power estimation algorithm utilizing the theoretical distribution of the alternative hypothesis was developed in this work and compared to classical Monte-Carlo studies. The parametric power estimation algorithm estimates the unknown non-centrality parameter in the theoretical distribution from a limited number of Monte-Carlo simulation and estimations. From the estimated parameter a complete power versus sample size curve can be obtained analytically without additional simulations, drastically reducing runtimes for this computation [17]. Further, type-I-error control in hypothesis testing with NLMEMs, can be implemented via permutation test [13, 18–21]. We established proof-of-principle examples how highly mechanistic systems pharmacology and/or systems biology models can be utilized in planning the analysis of clinical trials in small population groups. Based on simulations with the mechanism-based models more parsimonious models suitable for estimation can be utilized to understand drug effects and link to the mechanism-based model.

Model uncertainty is, for natural reasons, largest when based on estimation in a small sample size and at the same time a small sample size represents an extra challenge in accurately characterizing that uncertainty.

To assess parameter uncertainty distributions, sampling importance resampling constitutes a powerful alternative to estimate and utilize parameter uncertainty, especially in the context of small populations [22]. To this end, we developed diagnostics metrics to judge sampling importance resampling convergence.

Confidence intervals determined by bootstrap and stochastic simulation and re-estimation were compared. The bootstrap delta objective function value distribution provides an easy way to assess if bootstrap results in parameters contradicted by the original data [23]. Simulated and real data indicated that the bootstrap is often a sub-optimal method for imprecision estimates when the number of subjects is small, i.e. below around 100 subjects for standard pharmacokinetic data sets.

An automated preconditioning routine for NLMEMs to increase the computational stability of the variance-covariance matrix was developed. It demonstrated that the variance-covariance matrix and the R-matrix can give a strong indication on the non-estimability of the model parameters if computed correctly, while other methods may not be able to do so [24].

Model averaging methods were investigated in the case of dose selection studies (phase IIb). The proposed method reduces the analysis bias originating from the model selection bias of single model structure based analysis [25].

Model based adaptive optimal designs were investigated for bridging studies from adults to children, and were able to reduce model parameter uncertainty [26, 27].

*In summary, we developed new methods for sample size calculation, type I error control, model averaging and parameter precision in small populations group trials within non-linear mixed effects modelling.*

Recommendation 5. If fast computations of power curves are needed from a non-linear mixed effects model, we recommend using the parametric power estimation algorithm as implemented in the stochastic simulation and estimation tool of PsN (potentially with a type-I correction based on the “randtest” tool in PsN) [17, 20, 21].
Recommendation 6. The simulation methods described above can be utilized to investigate the effects of using different, smaller, more parsimonious models to evaluate data from complicated biological systems prior to running a clinical study [28, 29].
Recommendation 7. We recommend the use of Sampling Importance Resampling to characterize the uncertainty of non-linear mixed effects model parameter estimates in small sample size studies. Non-estimability of parameters may be assessed using preconditioning. The use of the bootstrap model averaging method [24] is recommended when conducting model-based decision-making after a trial. Robust model-based adaptive optimal designs may be used to improve model certainty in clinical trials [22–24, 27].

## Pharmacological consideration - optimal design

Optimal design techniques can be used to reduce the sample size by increasing the precision of the estimates in clinical trials providing longitudinal data. In the following we use optimal design methodology combined with adaptive design features to decrease the reliance on a priori assumptions. Particularly in rare diseases, repeated measures for each patient are most often available, at least to a certain extent. For instance, in model-based drug development, nonlinear mixed effects models are used to analyse the longitudinal data. Therefore, finding good designs for these studies is important to obtain precise results and/or good power especially when there are limitations on the sample size and on the number of samples/visits per patient. To answer the question of good or optimal designs in non-linear mixed effects modeling the variance of the model parameter estimates has to be optimized by means of the Fisher Information Matrix. This is particularly challenging when the study endpoint is discrete, of a repeated time-to-event nature, and with joint models. Here we developed two new methods to evaluate the Fisher Information Matrix. Both approaches first use Monte Carlo (MC) integration and then either Adaptive Gaussian Quadrature (MC-AGQ) [30] or Hamiltonian Monte Carlo (MC-HMC) [31]). Both approaches were evaluated and compared on four different examples with continuous, binary, count or time-to-event repeated data.

We showed the adequacy of both approaches in the prediction of the standard errors using clinical trial simulation. The MC-AGQ approach is less computational demanding for models with few random effects, whereas MC-HMC computational effort increases only linearly with the number of random effects, hence is more suitable for larger models. For both approaches we showed the importance of having large sampling number at the MC step. For the MC-AGQ method we illustrated for a binary outcome the influence of the design, i.e. the number of patients as well as the number of repetitions on the power to detect a treatment effect [30].

One limitation of the optimal design approach for NLMEs is the a priori knowledge needed about the parameter values. Adaptive design is a viable alternative, increasingly developed for randomized clinical trial or dose-ranging studies, but rarely applied in the context of NLMEMs. Two-stage designs are more practical to implement in clinical settings than fully adaptive designs, especially for small population groups.

We showed the good properties of adaptive two-stage designs when the initial guess about the parameters is wrong [32]. In the studied example, the efficiency of the balanced two-stage design was almost as good as a one-stage design that we would have obtained if the true parameters were known. With this small number of patients (*N* = 50), the best two-stage design was the balanced design with equal number of patients in each cohort. These results are consistent with those previously obtained [33] for a simpler example.

It is important to notice that model-based analysis of pivotal clinical trials in drug evaluation for small population groups allows for the use of all individual information recorded, and therefore for the decrease of sample sizes. One main limitation, as seen by health authorities, is the control of the type I error when performing model selection. Model averaging approaches offer a good alternative. The idea of pre-specifying a number of candidate models is already applied in drug development, for instance for dose-response studies in the MCPMod approach, but was extended only recently for mixed-effects models. Before the analysis step, one needs to design studies that are adequate across a set of candidate NLMEMs.

We proposed to use compound D-optimality criterion for designing studies that are robust across a set of pre-specified model. We also proposed robustness on the parameter values by defining prior distribution on each parameter and using the expected Fisher Information Matrix resulting in an MC-HMC method [34]. We evaluated those new developments on the count longitudinal data example where there is a model of the effect of dose on the Poisson parameter [30, 31, 34].

*In summary, we developed design evaluation methods enabling small clinical trials to be analysed through modelling of continuous or discrete longitudinal outcomes.*

Recommendation 8. For evaluation of designs of studies with longitudinal discrete or time-to-event data, evaluation of the Fisher Information matrix should be done without linearization. Using the new approach MC-HMC (in the R package MIXFIM) will provide adequate prediction of standard errors and allow to compare several designs [30, 31].
Recommendation 9. When there is little information on the value of the parameters at the design stage, adaptive designs can be used. Two-stage balanced designs are a good compromise. The new version of in the R functions PFIM can be used for adaptive design with continuous longitudinal data [33].
Recommendation 10. When there is uncertainty in the model regarding the parameters, a robust approach across candidate models should be used to design studies with longitudinal data [34].

## Pharmacological consideration - genetic factors

Another way to follow the advice of the CHMP guidance to use as much information as possible is to stratify patients according to assumed differential response to treatments. Stratification is of rapidly increasing interest in clinical research, in particular in personalized medicine [35] as well as in rare disease, since these diseases often have a stronger and simpler genetic causality. Modern drug development often aims at personalizing treatments; biomarkers are used to define subpopulations for which different treatments may be optimal. Nowadays, these biomarkers can be identified based on the high-dimensional “omics” (genomics, proteomics, metabolomics) data. However, to be effective for predicting the patients’ response to the treatment in small-population group trials this data needs to be preprocessed. The main purpose of this preprocessing is the reduction of dimensionality, so the number of parameters fitted when building the predictive model is smaller than the sample size. IDeAl proposed methods for reduction of dimensionality both for the whole genome genotype data as well as for highly correlated transcriptomics or metabolomics data. Specifically, the “group SLOPE” approach [36–38] for identification of important biomarkers based on the genotype data has been proved to be effective for identifying rare recessive genetic variants, which are particularly important in the context of rare diseases. On the other hand, the modified version of the Bayesian Information Criterion proposed in [39] allows to combine the genotype and ancestry data for an efficient identification of biomarkers in admixed populations. Concerning other types of “omics” data; the statistical package “varclust” [40] allows for identification of groups of highly correlated transcriptomics or/and metabolomics data. It can be used to identify genetic pathways related to the disease as well as for identification of a small number of principal components representing a given group of variables, which in turn can be used for building the predictive models. A new method “PESEL” [41] was proposed for selection of the number of relevant principal components. All these methods have been implemented in public available R packages.

Subsequently, a procedure for identifying the patients responsive to the treatment was proposed. It should be noted, that stratification can be implemented in the design phase, via inclusion criteria definition or as element of the randomisation process as well as in the analysis model. And of course, stratification could be a useful technique to increase the power of a trial in every setting.

*In summary, we developed new methods for identifying biomarkers and prognostic scores based on high dimensional genetic data in small population group trials.*

These developments lead to the following recommendations:

Recommendation 11. It is recommended to use “varclust” for clustering of gene expression or metabolomics data and extraction of a small number of potential predictors of patients’ response to the treatment based on highly dimensional “omics” [40]. Also, it is recommended to use PESEL for estimation of the number of important principal components [41].
Recommendation 12. It is recommended to use both regular and group SLOPE for identification of biomarkers based on the genotype data, since regular SLOPE has a higher power of detection of additive gene effects, while group SLOPE allows for identification of rare recessive variants [37].
Recommendation 13. It is recommended to use the modified Bayesian Information Criterion for efficient aggregation of genotype and ancestry of genetic markers and identifying biomarkers in admixed populations [39].

## Choice of endpoint - biomarkers

Definition of a suitable endpoint to measure or assess the benefit of a new treatment is a central point in clinical-trial design. The importance of the definition of suitable endpoints in rare disease clinical trials is already mentioned in the CHMP guideline and further discussed by the IRDiRC report on Patient-Centred Outcome Measures 2016 [42]. In particular, in rare diseases, there is a need for quickly accessible endpoints, for instance in cases when the limited patient population size makes it infeasible to use, for example, dichotomous therapeutic outcomes as the primary variable in confirmatory trials. Thus an efficient and feasible framework to evaluate biomarkers and surrogate endpoints in small population group clinical trials was needed. This development includes various aspects like handling of missing-data, design aspects like randomisation methodology, optimal design, adaptive designs, decision theory, hierarchical-data models, cross-over trials as well as incorporating genetic markers and dose response information.

We showed that for small-populations groups, a causal inference framework is especially useful [43–45]. Further, to account for missing data, the use of pseudo-likelihood and inverse probability weighting methods are shown to be advantageous over commonly used full pseudo-likelihood methods while validation of surrogate endpoints [46, 47]. Efficient and stable estimation strategies for the validation model which of course could be non-linear as well are developed [48]. Another aspect which is important in drug discovery is the use of high-dimensional biomarkers [49]. Further dose-response information is extremely valuable in the context of markers in general and surrogate endpoints in particular [50].

When surrogate markers are evaluated, the use of multiple units (centres, trials, etc.) is needed, no matter which paradigm is used. It is well-known that full likelihood estimation is usually prohibitive in such complex hierarchical settings, in particular when trials are of unequal (and small) sizes. This phenomenon has been examined by [51]. Based on this we propose solutions for simple but generic longitudinal settings with units of unequal size; these solutions are based on weighting methods.

*In summary, we developed a methodology for evaluating potential surrogate markers and to analyse data from a small numbers of small trials, with emphasis on fast and easy computational strategies.*

This leads to the following recommendations in the context of evaluation of biomarkers or surrogate endpoints in small population clinical trials:

Recommendation 14. In case of small trials, which are in particular variable in size, we recommend the use of the causal inference framework, combined with efficient computational methods [43–45].
Recommendation 15. In case of the evaluation of surrogate endpoints in small trials subject to missingness, we recommend the use of pseudo-likelihood estimation with proper inverse probability weighted and doubly robust corrections [46, 52].
Recommendation 16. In case of hierarchical and otherwise complex designs, we recommend using principled, yet fast and stable, two-stage approaches [51].
Recommendation 17. In case of genetic and otherwise high-dimensional markers, we recommend the use the methodology expressly developed for this context, in conjunction with the software tools made available (R package IntegratedJM) [49, 50].
Recommendation 18. In case of a surrogate with dose-response or otherwise multivariate information present, we recommend to use the Quantitative Structure Transcription Assay Relationship framework results. [50].
Recommendation 19. In case of the evaluation of surrogate endpoints in small studies, we recommend using weighting-based methods, because the methodology has been shown to work well theoretically, because it has been implemented in user-friendly SAS and R software, and because its practical performance is fast and stable [48, 49, 51]

Among other aspects to validate a clinical endpoint reliability, i.e. the correlation between repeated measurements that are taken within the same subject is of major interest [42]. For example, the same outcome may be measured repeatedly over time in the same patients. In practical settings, the estimation of reliability become more complex by the design under investigation. We propose a general and flexible modelling approach to estimate reliability, as well as the standard errors, and confidence intervals [53].

## Methodological considerations - randomisation

An important design technique used in comparative clinical trials is randomisation, i.e. the treatment allocation by an element of chance. This technique is applied in almost all confirmatory clinical trials, where two and more treatments are compared to each other. Here the element of chance in the allocation process is used to avoid or at least minimize the influence of bias on the estimate of the treatment difference. The properties of randomisation procedures are well understood from the theoretical point of view, but little work has been done with respect to practical situations. For instance, apart from response adaptive randomisation procedures, the direct impact of randomisation on the endpoints is under-investigated. Further, most of the evaluations belong to the long run argument, which is hardly applicable in small clinical trials. On the other hand, the choice of the randomisation procedure for a particular clinical trial is generally up to the scientist “feeling” and frequently not well motivated by scientific arguments. We showed that false decisions for a treatment effect can be caused by failure to select the best practice randomisation procedure. To assess the value of randomisation procedures for designing small clinical trials, a completely new methodology had to be developed. IDeAl implements rigorously the relation of the randomisation process to the endpoint. The model for selection bias as well as time trend bias can be interpreted as covariance imbalance and thus has strong relation to stratification.

In various papers we developed a mathematical model to describe the impact of selection bias on the type-I-error probability for two- [54] and multi-arm [55] parallel group designs with continuous normal endpoint as well as for time-to-event endpoints [56]. We showed that the impact is more heterogeneous in smaller trials than in larger trials.

We investigated the impact of time trend of different forms [57] and included this in the models above. We developed a linked assessment criterion, based on a normalized multi-criterion function [58] to be able to investigate various purposes. All these derivations are included in our proposed evaluation of randomisation procedures to clinical trial design optimization (ERDO) framework, which will lead to more rational randomized patient allocation procedures, giving trial results that are more robust to selection bias and to inflation of the conditional type-I-error rate [59]. ERDO should be used as part of the clinical trial planning. The framework makes use of our R package randomizeR [60]. We reached to the conclusion, that no randomisation procedure protects against all types of bias in every clinical situation, however some perform better than others. Consequently, we advocated for a bias-corrected hypothesis test. We developed an asymptotic likelihood ratio test to analyse randomized clinical trials that may be subject to selection bias for normally distributed responses [61]. Other options are inclusion of the block factor when only time trend affects the data [57] as well as modelling [55]. These analyses should be part of the sensitivity analysis of a clinical trial to assess the level of evidence.

*To sum up, we developed a new methodology for the selection of the best practice randomisation procedure and subsequent analysis for a small population clinical trial taking possible bias into account.*

This leads to the following three recommendations:

Recommendation 20. Do not select a randomisation procedure by arbitrary arguments, use scientific arguments based on the impact of randomisation on the study endpoint taking into account the expected magnitude of bias [54–57].
Recommendation 21. Tailor the randomisation procedure used in small-population randomized clinical trial by following ERDO using randomizeR [59, 60].
Recommendation 22. In case of a randomized clinical trial, we recommend to conduct a sensitivity analysis to examine the impact of bias on the type-I-error probability [55, 59–62].

It should be noted, that the findings about the validity of randomisation should be applied to every clinical trial design used in small population clinical trials, see below. The consequence is a better understanding about the evidence, which could be expected or is derived from a clinical trial. Currently the ERDO is applied to several studies, for instance the NICOFA trial to study Nicotinamide for the treatment of Friedreich ataxia with principal investigator Jörg Schulz (Chair of Department of Neurology, University Clinic Aachen, http://www.erare.eu/all-funded-projects).

## Methodological considerations - adaptive design

Adaptive design techniques have been widely discussed over the last decades [63, 64] and in particular appear in the context of small population clinical trials very promising [65]. IDeAl used adaptive design techniques in connection with extrapolation as well as optimal design techniques, see above.

The use of external information in designing and analyzing clinical trial data has attracted much interest and it is supposed that this fasten the validation process of new therapies. There are several areas, which might be promising here. For instance, the use of historical data to substitute parts of a randomized trial, the extrapolation of knowledge from one disease population to another as well as the acceptance of already derived knowledge from single arm trials so that further trials are not necessary. IDeAl considers the problem of using rigorously the data from a single arm study, using the data from a previous trial to adapt the trial in a small population and extrapolation of a dose response curve.

Another way to incorporate external information in the design and/or the analysis of a clinical trial is introduced by Hlavin [66]. The method used the strength of the current knowledge in a large population or for instance in adults to modify the significance level of the clinical trial in the small population, i.e. children. Of course, by this the sample size in the trial in the smaller population can be decreased. The approach makes use of Bayesian arguments to formulate a scepticism factor which reflects the confidence in the actual knowledge. This approach seems promising in pediatric trials to implement an adaptive pediatric investigation plan [5].

Of course, a point to consider when using external information is related to sharing clinical-trial data at patient level. Not only the data protection problem should be taken into account, but also the statistical problem related to post-hoc analysis. Expertise in biostatistics is needed to assess the interpretation of such multiple analyses, for example, in the context of regulatory decision-making by application of optimizing procedural guidance and sophisticated analysis methods [67].

In the ICH E10 guideline [68], it is mentioned that it may be tempting in exceptional cases to initiate an externally controlled trial, hoping for a convincingly dramatic effect, with a prompt switch to randomized trials if this does not materialize. This leads to the idea of the new framework, i.e. “threshold-crossing”, which leverages the wealth of information that is becoming available from completed RCTs and from real world data sources [69]. The main idea is to formulate a threshold to be applied in a single arm trial, which serves as a decision rule for the need of a randomized trial.

Testing for multiple objectives in clinical trials is preferable, while supposed to reduce the number of clinical trials and thus affects all clinical trials. However, if the type I error probability is not considered accordingly, a conflict with the validity of the statistical analysis arises. The problem becomes more challenging with combining multiple objectives with adaptive design techniques. We developed adaptive graph-based multiple testing procedures to allow testing of multiple objectives and designs adaptations in a confirmatory clinical trial [70]. The methodology is applicable in a wide range of scenarios including trials with multiple treatment comparisons, endpoints or subgroups, or combinations thereof. If, in the interim analysis, it is decided to continue the trial as planned, the adaptive test reduces to the originally planned multiple testing procedure. Only if adaptations are actually implemented, an adjusted test needs to be applied.

We considered Phase IIb dose finding studies. To plan and analyse these studies the European Medicines Agency has qualified the MCP-Mod approach. Originally MCP-Mod was developed for Phase IIb dose finding studies to characterize the dose response relationship under model uncertainty once a significant dose response signal has been established. We developed a new closed MCP-Mod methodology for confirmatory clinical trials to allow individuals claims that a drug has a positive effect for a specific dose and applied the closed MCP-Mod methodology to adaptive two-stage designs by using an adaptive combination tests.

In a recent review conducted by the European Medicines Agency [71] it was shown that most of the adaptive design proposals were in oncology. Unfortunately, the important case of time-to-event endpoints is not easily handled by the standard adaptive theory. We proposed an alternative frequentist adaptive test which allows adaptations using all interim data [72]. We showed that other standard adaptive methods may ignore a substantial subset of the observed event times. Further, we developed a group sequential permutation test for situations where the underlying censoring mechanism would be different between the treatment groups [73].

*To summarize at this point, we developed statistical methods to adapt the significance level and allow confirmatory decision-making in clinical trials with vulnerable, small populations.*

Recommendation 23. In the case of confirmatory testing, we recommend adapting the significance level by incorporating other information, e.g. using information from drug development programs in adults for designing and analyzing pediatric trials [66].
Recommendation 24. Where randomized control clinical trials are infeasible, we propose “threshold-crossing” designs within an adaptive development program as a way forward to enable comparison between different treatment options [69].
Recommendation 25. In the case of design modification during the conduct of a confirmatory clinical trial, we recommend using adaptive methods to ensure that the type-I-error is sufficiently controlled not to endanger confirmatory conclusions. Especially in clinical trial with multiple objectives special care has to be taken to address several sources of multiplicity [70].

## Methodological considerations - pharmacogenetics

IDeAl investigated various special designs. For instance, statistical design considerations in first in human studies, which usually are supposed to be of small size, and are necessary in all drug development programs were discussed in [74]. The six key issues highlighted in the paper are dose determination, availability of pharmacokinetic results, dosing interval, stopping rules, appraisal by safety committee, and clear algorithm required if combining approvals for single and multiple ascending dose studies.

We developed approaches to planning and analyzing trials for identifying individual response to treatment effects in small populations from various perspectives.

Crossover designs, as an extension of N-of-1 trials, can be used to evaluate between and within subject variability. This is particularly of interest in personalized medicine where a repeated crossover design is suitable for identifying variability arising between treatments and from interaction between individual patients and their treatment [35]. However, the lack of standards for reporting clinical trials using a crossover layout is mentioned in particular for evaluation of analgesic treatment for chronic pain [75].

The N-of-1 trial design is of special interest for IDeAl, in particular because such designs may be particularly suited to proof of concept studies. More generally, the research design should reflect a specific research question. For example, if the intention is to determine efficacy of a treatment for a single patient, the N-of-1 trial design is recommended in chronic diseases [75]. However, such trials can also be extremely efficient and thus N-of-1 trials can be particularly useful in small populations [1]. Two purposes of the analysis of an N-of-1 trial are establishing whether a treatment works at all and establishing to what extent the effect varies from patient to patient. Here the evaluation of the within patient variability becomes of major interest. Of course, the latter can only be answered if a series of N-of-1 trials is available. We demonstrated that the appropriate analysis employed could vary radically according to the questioned it was desired to answer [76].

When designing an N-of-1 trial, an important question concerns the samples size. When concentrated on addressing the challenge of N-of-1 trials, there are many components of variation involved, which make sample size determination complex. We developed methods reflecting these challenges and also the different questions that may be addressed [77].

An important aspect in the analysis of clinical trials with small population groups is the explanation of the sources of variation. For example, for longitudinal trials both within- and between-patient variation have to be considered as a minimum. If treatments are varied within the same patient other sources of variation have to be included. This shows that the trial design is a key element in the discussion of various sources of variation in observed response in clinical trials. It is suggested that reducing variation in medical practice might make as big a contribution to improving health outcome as personalizing its delivery according to the patient. It is concluded that the common belief that there is a strong personal element in response to treatment is not based on sound statistical evidence.

In rare diseases, it is even more important and promising than in larger trials to extract as much information as possible from between-patient trials. This has been addressed trough a number of ways, e.g. making efficient use of covariates. We explored machine learning techniques, where the number of values of a response variable can be very high and reducing the values by clustering improves performance. The aim is to formulate a prediction model, i.e. select appropriate covariates. We developed an algorithm that simultaneously groups the values of a response variable into a limited number of clusters and selects stepwise the best covariates that discriminate this clustering [78]. However, care has to be taken to the selection of the covariates.

*In summary, we developed approaches to planning and analyzing trials for identifying individual response and examining treatment effects in small populations.*

Recommendation 26. For the analysis of N-of-1 trials, we recommend using an approach that is a modified fixed-effects meta-analysis for the case where establishing that the treatment works is the objective, and an approach through mixed models if variation in response to treatment is to be studied [76].
Recommendation 27. When conducting a series of N-of-1 trials we recommend paying close attention to the purpose of the study and calculating the sample size accordingly using the approach provided in detail in Senn [77].
Recommendation 28. We recommend that response should not be defined using arbitrary and naïve dichotomies but that it should be analysed carefully paying due attention to components of variance and where possible using designs to identify them [79].
Recommendation 29. When analyzing between-patient studies, we recommend avoiding information-destroying transformations (such as dichotomies) and exploiting the explanatory power of covariates, which may be identified from ancillary studies and patient databases.

## Extrapolation

As stated in the CHMP guidance [1] it is recommended to use as much information as possible to improve the design. IDeAl follows this advice extensively, and as one first aspect investigated options and methods for extrapolation.

In this context regression models are a very important tool to provide dose-response information. In many cases the question occurs whether two dose response curves can be assumed to be identical. This problem also appears in the situation of detecting non-inferiority and/or equivalence of different treatments [80].

We derived new statistical procedures addressing the problem of comparing curves and extrapolating information, with a particular focus on trials with small sample sizes.

We improved the previous standard, less powerful methodology for comparing two such curves [81], and showed that the efficiency can be considerably increased using a bootstrap approach. Additionally, we developed a new statistical test for the hypothesis of similarity of dose response curves. The test decides for equivalence of the curves if an estimate of a distance is smaller than a given threshold, which is obtained by a (non-standard) constrained parametric bootstrap procedure [47]. A corresponding R package “TestingSimilarity” was developed [82].

Further the Minimum Effective Dose (MED) metric [83] was used to measure for similarity of dose-response by claim for equivalence (to a certain amount) of information from the source and the target population. Confidence intervals and statistical tests were developed for this metric [84]. We further could show the very robust performance of all derived methodologies [85]. Finally, optimal designs for the comparison of curves have been developed, which minimizes the maximum width of the confidence band for the difference between two regression functions. In particular, it was demonstrated that the application of optimal designs instead of commonly used designs yields a reduction of the width of the confidence band by more than 50% [86, 87].

*In summary, we developed a new optimized design and analysis strategy for comparing dose-response profiles to extrapolate clinical trial results from a larger to a smaller population.*

Recommendation 30. The comparison of dose response curves should be done by the bootstrap approach [47, 87].
Recommendation 31. If the aim of the study is the extrapolation of efficacy and safety information, we recommend considering and comparing the MEDs of two given populations [84].
Recommendation 32. The derived methodology shows a very robust performance and can be used also in cases where no precise information about the functional form of the regression curves is available [88].
Recommendation 33. In case of planning a dose-finding study comparing two populations, we recommend to use optimal designs in order to achieve substantially more precise results [86, 87].

As a perspective, it should be noted, that the extrapolation can be combined with the surrogate endpoint findings. For instance, if the dose-response curve is established in adults following a true endpoint, and there is already a validated surrogate endpoint in a pediatric population available, then the latter can be used to show similarity of the dose-response curves.

## Software

In the preceding sections we discussed various aspects to improve the design and analysis of small population clinical trials ending up the 33 recommendation. However, software packages are necessary to implement these recommendations. Various software packages have been delivered, to facilitate the application of our findings. The lists can be found as supplementary material in Table 1. More information can be found on the website (https://www.ideal.rwth-aachen.de/).

**Table 1:** List of IDeAl Statistical Software

1. Araujo, A. (2016): *R-Code “Statistical Analysis of Series of N-of-1 Trials Using R”*, http://www.ideal.rwth-aachen.de/wp-content/uploads/2014/02/nof1_rand_cycles_v8.pdf	
2. Brzyski, D. Peterson, C., Candes, E.J., Bogdan, M., Sabatti, C., Sobczyk, P. (2016): *R package “geneSLOPE”* for genome-wide association studies with SLOPE. https://cran.r-project.org/web/packages/geneSLOPE/index.html	
3. Graf, A., Bauer, P., Glimm, E., König, F. (2014): *R-Code to calculate worst case type I error inflation in multiarmed clinical trials*, http://onlinelibrary.wiley.com/doi/10.1002/bimj.201300153/suppinfo	
4. Jobjörnsson, S. (2015): *R package “bdpopt”* for optimization of Bayesian Decision Problems. https://cran.r-project.org/web/packages/bdpopt/index.html	
5. Hlavin, G. (2016): *application for extrapolation to adjust significance level based on prior information*, http://www.ideal-apps.rwth-aachen.de:3838/Extrapolation/	
6. Möllenhoff,K. (2015): *R package “TestingSimilarity”* for testing similarity of dose response curves. https://cran.r-project.org/web/packages/TestingSimilarity/	
7. Riviere, M.K., Mentré, F. (2015): *R package “MIXFIM”* for the evaluation and optimization of the Fisher Information Matrix in Non-Linear Mixed Effect Models using Markov Chains Monte Carlo for both discrete and continuous data. https://cran.r-project.org/web/packages/MIXFIM/	
8. Schindler, D., Uschner, D., Manolov, M, Pham, M., Hilgers, R.-D., Heussen, N. (2016): *R package “randomizR”* on Randomization for clinical trials. https://cran.r-project.org/web/packages/randomizeR/	
9. Senn, S, (2014): *R, GenStat and SAS Code for Sample Size Considerations in N-of-1 trials*, http://www.ideal.rwth-aachen.de/wp-content/uploads/2014/02/Sample-Size-Considerations-for-N-of-1-trials.zip	
10. Sobczyk, P., Josse, J., Bogdan, M. (2015): *R package “varclust”* for dimensionality reduction via variables clustering. https://psobczyk.shinyapps.io/varclust_online/	
11. Sobczyk, P., Josse, J., Bogdan, M. (2017): *R package “pesel”* Automatic estimation of number of principal components in PCA with PEnalized SEmi-integrated Likelihood (PESEL). https://github.com/psobczyk/pesel	
12. Szulc, P., Frommlet, F., Tang, H., Bogdan, M. (2017): *R application for joint genotype and admixture mapping in admixed populations*, http://www.math.uni.wroc.pl/~mbogdan/admixtures/	
13. Van der Elst, W., Alonso, A., Molenberghs, G. (2017): *R package “EffectTreat”* on the Prediction of Therapeutic Success. https://cran.r-project.org/web/packages/EffectTreat/index.html	
14. Van der Elst, W., Meyvisch, P., Alonso, A., Ensor, H.M., Weir, C.J., Molenberghs, G. (2017): *R Package “Surrogate”* for evaluation of surrogate endpoints in clinical trials. https://cran.r-project.org/web/packages/Surrogate/	
15. Van der Elst, W., Molenberghs, G., Hilgers, R.-D., Heussen, N. (2016): *R package “CorrMixed”* for the estimation of within subject correlations based on linear mixed effects models. https://cran.r-project.org/web/packages/CorrMixed/index.html	

## IDeAl-net

We derived 33 recommendations from IDeAl’s more than 60 published scientific papers to date in peer-reviewed journals, to improve the design and analysis of small population clinical trials. The results belong to general aspects of clinical trial design and analysis methods as well as to more specific areas. General techniques include methodologies at a drug development level like decision theoretic evaluation as well as at the trial design level like choice of a randomisation procedure, establishing surrogate endpoints, development of prognostic factors, pharmacometric evaluation of design aspects, adaptive graph-based multiple testing procedures. Special techniques address for instance dose response trials with respect to extrapolation, designs for longitudinal data. Of course, application of these recommendations depends on the practical situation, e.g. the disease under investigation etc. The importance of advanced statistical modelling becomes clearer with the application in the rare disease context. For instance, mixed effects models, whether linear or non-linear constitute important statistical methodology, not only for evaluation of surrogate endpoints, for analysis of pharmacological considerations, but also for the analysis of subject by treatment interaction as in personalized medicine [88]. Further, the definition of an endpoint as slope over time is another area of successful application of linear mixed-effects models which reduces the sample size [89].

**Fig. 1:**
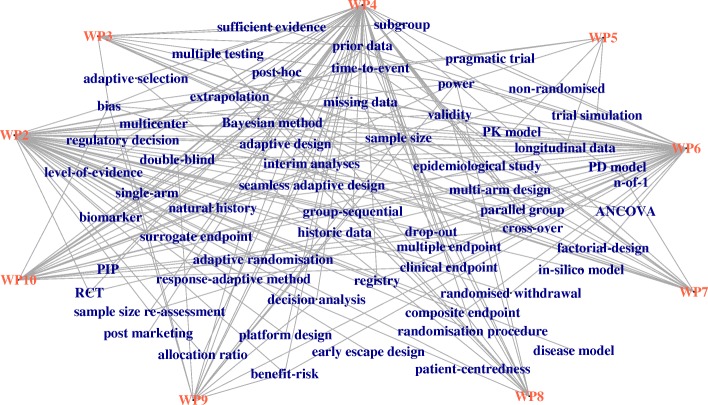
IDeAl-net-1 relating IRDiRC task force report design and analysis topics to IDeAl’s work package output

Some researcher might prefer to think in terms of special topics representing methodological skills necessary to design small population clinical trials and would like to mirror IDeAl’s work on these topics. A sound list of topics, which are currently discussed in the area of improvement of the statistical design and analysis methodology for small population clinical trials is summarized in the IRDiRC task force report [65]. We extracted a list of 73 items (see supplementary material for a complete list in Table 2) related to statistical design and analysis methods from this report. To relate the work package output to these IRDiRC task force report items we conducted a “text mining” search in the 65 IDeAl publications. 12 topics are not addressed by IDeAl’s work. This result is illustrated as network graph expressing the topics reflected by IDeAl’s research (see Fig. 1: The **IDeAl-net-1**).

**Table 2:** List of IRDiRC task force report design and analysis topics and synonyms (topics in italics are not addressed in IDeAl’s publications)

adaptive design; adaptive/flexible design/study/trial	
adaptive randomisation	
adaptive selection	
allocation ratio	
ANCOVA	
Bayesian method; method/analysis/design	
benefit-risk	
bias	
biomarker; bio/genetic	
clinical endpoint; endpoint/outcome	
composite endpoint; endpoint/outcome/measure/response measure	
cross-over	
decision analysis, analysis/theory/making/process	
*disease mechanism*	
disease model	
double-blind	
drop-out	
*drug-disease model; model/modelling*	
early escape design	
*enhanced trial design*	
epidemiological study	
extrapolation	
factorial-design	
group-sequential	
*high-risk allocation design*	
historic data	
in-silico model; model/modelling/clinical trial	
interim analyses	
level-of-evidence	
longitudinal data; longitudinal/repeated measures, model/data/outcome	
*micro-dose trial; trial/study*	
missing data; midding data/missingness	
multi-arm design; multi arm/multiple treatment arm, design/study	
multicenter	
multiple endpoint; endpoint/outcome	
multiple testing; multiple testing/multiple hypotheses testing	
natural history	
n-of-1; n-of-1/single-subject design	
*non-clinical data*	
non-randomised	
parallel group	
*patient preference trial*	
patiet-centerdness, centerdness/centered	
*PCOM;* *patient-centered* *outcome measures*	
PD model; PD/pharmacodynamic	
PIP; paediatric investigation plan	
PK model; PK/pharmacokinetic	
platform design; design/trial	
post marketing	
post-hoc	
power	
pragmatic trial; trial/study	
prior data; data/distribution, informative Bayesian prior distribution	
*prognostic model; model/factor*	
*public health strategy*	
randomisation procedure	
randomised withdrawal	
RCT; randomised controlled trial/study/design	
registry	
regulatory decision; decision/strategy	
*re-randomisation*	
response-adaptive method; method/design	
sample size	
sample size re-assessment; reassessment/re-estimation	
seamless adaptive design	
single-arm	
*SMART design; SMART/snSMART*	
subgroup; group/population	
sufficient evidence	
surrogate endpoint; endpoint/outcome/marker	
time-to-event; survival endpoint/outcome/trial/study	
trial simulation	
validity

**Fig. 2:**
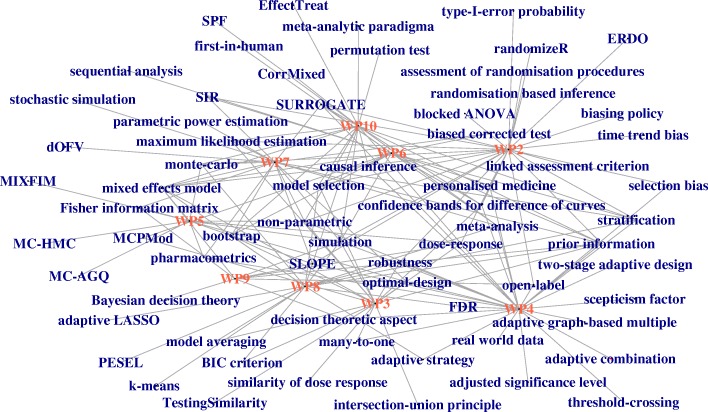
IDeAl-net-2 relating a list of statistical techniques relating to IDeAl’s work package outputs

On the other hand, IDeAl’s findings make use of specific statistical skills and introduce new methods beyond the IRDiRC task force report. To design and analyse a small population groups trial “IDeAl-ly” the terms included in the IDeAl-net-2 should be taken into account. Again IDeAl-net-2 is based on the 65 IDeAl publications relating the work package output to terms newly coined terms (see Fig. 2: The **IDeAl-net-2** supplementary material for a complete list in Table 3). The graphs not only illustrate how the topics are related to the work package tasks but also how the topics are related to each other. This shows that design aspects are related at various levels as pointed out in the IRDiRC task for report [3, 68]. Inspired by the unidirectional graph presented by Cornu et al. [90] we developed a more complex graphical representation of design and analysis methods necessary to tailor small population clinical trials.

**Table 3:** List of IDeAl added aspects, explanation in brackets

adaptive combination	
adaptive graph-based multiple	
adaptive LASSO	
adaptive strategy	
adjusted significance level	
assessment of randomisation procedures	
Bayesian decision theory	
biased corrected test (likelihood ratio test)	
biasing policy	
BIC criterion	
blocked ANOVA	
bootstrap (constrained parametric bootstrap procedure)	
causal inference	
CorrMixed	
decision theoretic aspect (Bayesian decision theoretic)	
dOFV (delta objective function values)	
dose-response	
EffectTreat	
ERDO (evaluation of randomisation procedures for design optimisation)	
similarity of dose response	
FDR (false discovery rate)	
first-in-human	
Fisher information matrix	
SLOPE (group SLOPE, geneSLOPE)	
k-means	
linked assessment criterion	
many-to-one	
maximum likelihood estimation	
MC-AGQ	
MC-HMC	
MCPMod (closed MCPMod)	
confidence bands for difference of curves	
meta-analysis	
meta-analytic paradigma	
mixed effects model	
MIXFIM	
model averaging	
model selection	
monte-carlo	
stochastic simulation	
non-parametric	
open-label	
optimal-design (compound D-optimality criterion)	
parametric power estimation	
permutation test	
PESEL (penalized semi-integrated likelihood method)	
pharmacometrics	
prior information	
randomisation based inference	
randomizeR	
real world data	
Robustness	
SIR (sampling importance resampling)	
scepticism factor (scepticism)	
selection bias	
sequential analysis	
Simulation	
SPF (surrogate predictive function)	
stratification	
personalised medicine	
SURROGATE	
TestingSimilarity	
threshold-crossing	
time trend bias	
two-stage adaptive design	
type-I-error probability	
intersection-union principle	

## Discussion

As described in the previous chapters, IDeAl has contributed to the most important areas of statistical design and analysis of small population clinical trials with a significant number of new results. This already refines the actual methodologies. However, it is shown that major progress is being made, which not only improves the standard methods dramatically [3, 91]. Some of the findings, like the adaptive extrapolation with refining the significance level, the recommendation about the selection of a randomisation procedure as well as the decision analytical approach necessitate rethinking, flexibility of stakeholders and thus braking barriers is necessary.

**Fig. 3:**
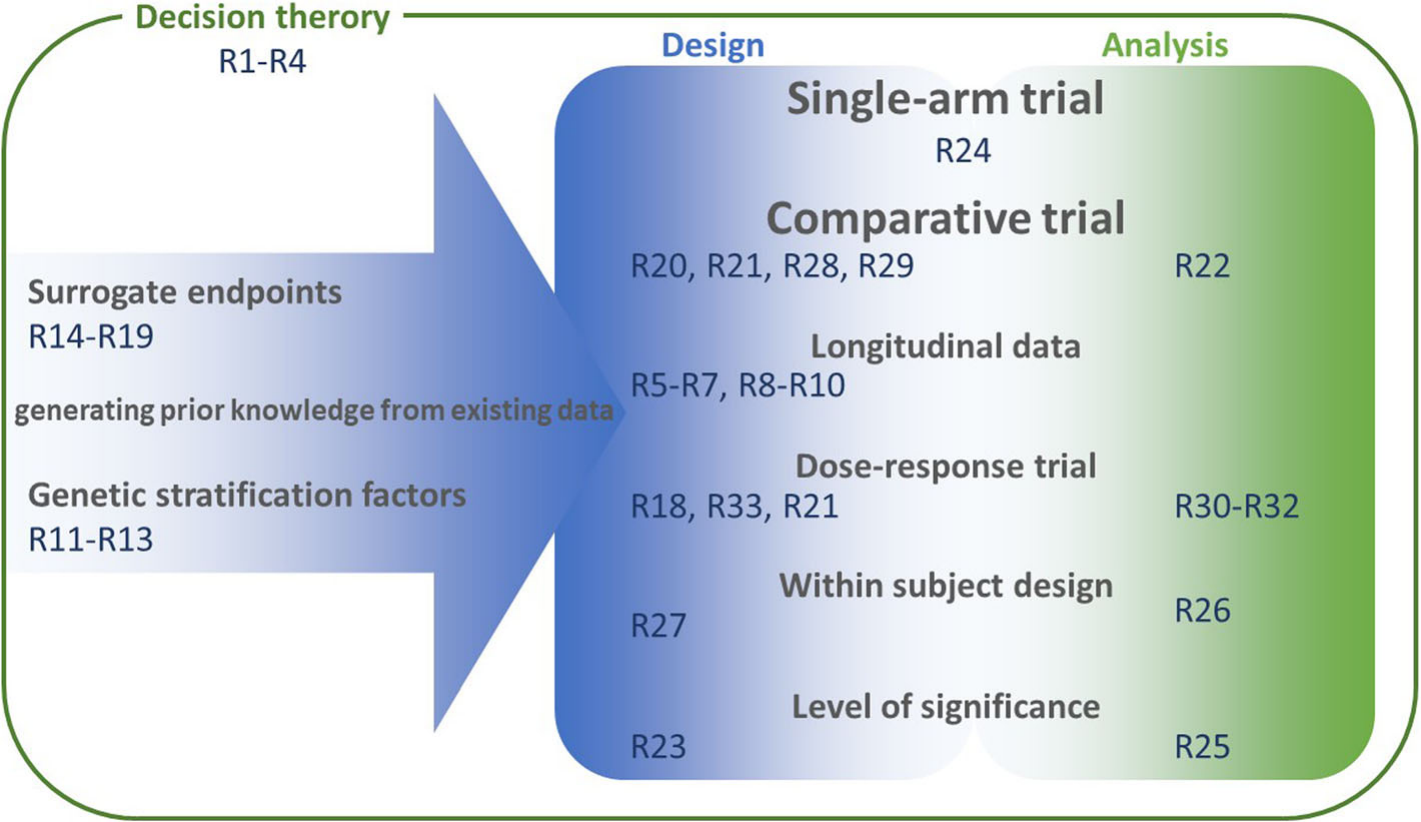
IDeAls recommendation related to planning clinical trials

To give research a direction how to use the recommendations, we refer to Fig. 3. From the point of view to plan a clinical trial, some recommendations belong to generating information form existing data while other belong to the integrated design and analysis perspective. The overaching assessment about the value of a research program making use of decision theoretic is addressed as well.

Of course, this is only a report after 3 year of developments. Further research is already started going far beyond the initial IDeAl research plans and will add new aspects. Some of these further results have already been summarized in scientific publications that are under review. Some other work is still in progress with papers under preparation. These aspects are not mentioned in the paper here. However, the IDeAl consortium feel that the actual description included in the paper is worth to report in the light of an expected review of the CHMP guidance on small population clinical trials next year. Here this report is already helpful to define one side of a new standard. Of course, the forthcoming results of asterix and InSPiRe, are the other side and are an excellent basis for new arguments as well as the results from the before mentioned projects under investigation. Some of the developed procedures have the potential to become a certified procedure [92].

IDeAl already shows the relation to other research areas that might seem far away from small population groups. The bridge between big data and small-population clinical trials was built up resulting in recommendations for an European Union action plan in [93].

A total of 33 recommendations developed and related to the work packages are given. The route to thinking about improvements is displayed in an IDeAl-network, which is grounded on IRDiRC topics which are discussed in the context of small population clinical trials. This shows in particular, that unfortunately, there is no “one size fits all” solution and as a result of IDeAl research, one may conclude that tailored approaches are necessary for statistically designing and analyzing small population group trials. Here experts are necessary to train different stakeholders. Teams, perhaps including more than one biostatistician should be formed to answer specific questions.

## Conclusion

To date, IDeAl has brought major progress to the designs and analysis of small population clinical trials. Some of the findings concern all areas of clinical trials while other address specific research questions. With this, evidence can be derived in small population clinical trials. The methods can be used in a wide range of small population clinical scenarios. Rigor and thoughtful application will offer opportunities in clinical scenarios where trials are infeasible with the standard methods.

## Abbreviations

asterix: Advances in Small Trials dEsign for Regulatory Innovation and eXcellence
CHMP: Committee for Medicinal Products for Human Use
EMA: European Medical Agency
ERDO: Evaluation of Randomisation procedures to clinical trial Design Optimization
EU: European Union
FDA: U.S. Food and Drug Administration
IDeAl: Integrated Design and Analysi of small population group trials
IDeAl-net: Design and Analysis Network developed by IDeAl
InSPiRe: Innovative methodology for small populations research
IRDiRC: International Rare Disease Research Consortium
MC: Monte Carlo
MC-AGQ: Monte Carlo Adaptive Gaussian Quadrature
MC-HMC: Monte Carlo Hamiltonian Monte Carlo
MCPMod: Multiple Comparison Procedures – Modelling
MED: Minimum Effective Dose
MIXFIM: Evaluation of the FIM in NLMEMs using MCMC
NLMEM: Nonlinear mixed-effects models
PCOM: Patient Centred Oucome Measures
PD: Pharmacodynamic
PFIM: Population Fisher information matrix
PIP: Peadiatric Investigation Plan
PK: Pharmakokinetic
PsN: Perl-speaks-NONMEM
R: Statistical software
R&D: Research and development
RCT: Randomized Controlled Trial
